# Dr. Newton Freeman Curtis

**Published:** 1914-06

**Authors:** 


Dr. Newton Freeman Curtis, P. & S., 1874; died in Milton, Mass., April 30, aged 64. He was a practitioner of White Plains, N. Y. for many years.

